# Medical Society

**Published:** 1846-12

**Authors:** 


*Medical Society.*—A meeting of the County Medical Society was held on Tuesday, October 13th, to consider the propriety of petitioning the Legislature, to abolish all the laws relating to Medical Societies. Unusual pains were taken to get a full meeting of the Societ y and an expression of deliberate opinion upon the novel and important proposition. At the hour of meeting, a large number of members were assembled, Dr. Isaac Wood in the chair. The resolutions were introduced by Dr. A. Wright, who advocated them with great earnestness. The Society was afterwards addressed by Dr. Stearns, (who gave some very interesting reminiscences of the doings of the County Medical Societies in other days, and of the good influence their establishment exerted upon the then condition of the profession,) Drs. Manley, Sheerwood, Childs, Beadle and others. The question was finally taken by yeas and nays, and the proposition negatived by a large majority. From this it appears, that the profession are not prepared to abandon their present organization.—*New York Annalist.*

